# Identification of immune related molecular subtypes and prognosis model for predicting prognosis, drug resistance in cervical squamous cell carcinoma

**DOI:** 10.3389/fgene.2023.1137995

**Published:** 2023-03-17

**Authors:** Dongzhi Hu, Zijian Zhang, Yongjing Zhang, Kangni Huang, Xiaoxue Li

**Affiliations:** ^1^ Department of Obstetrics and Gynecology, Yiyang Central Hospital, Yiyang, China; ^2^ Department of General Surgery, The Second Xiangya Hospital of Central South University, Changsha, China; ^3^ Department of Obstetrics and Gynecology, The Second Xiangya Hospital of Central South University, Changsha, China

**Keywords:** tumor microenvironment, endocervical adenocarcinoma, immunotherapy, genomic mutations, prognosis

## Abstract

**Background:** One of the features of tumor immunity is the immunosuppressive tumor microenvironment (TME). In this study, TME gene signatures were used to define the characteristics of Cervical squamous cell carcinoma (CESC) immune subtypes and construct a new prognostic model.

**Methods:** Single sample gene set enrichment analysis (ssGSEA) was used to quantify pathway activity. RNA-seq of 291 CESC were obtained from the Cancer Genome Atlas (TCGA) database as a training set. Microarray-based data of 400 cases of CESC were obtained from the Gene Expression Compilation (GEO) database as an independent validation set. 29 TME related gene signatures were consulted from previous study. Consensus Cluster Plus was employed to identify molecular subtype. Univariate cox regression analysis and random survival forest (RSF) were used to establish the immune-related gene risk model based on the TCGA data set of CESC, and the accuracy of prognosis prediction was verified by GEO data set. ESTIMATE algorithm was used to perform immune and matrix scores on the data set.

**Results:** three molecular subtypes (C1, C2, C3) were screened in TCGA-CESC on account of 29 TME gene signatures. Among, C3 with better survival outcome had higher immune related gene signatures, while C1 with worse prognosis time had enhanced matrix related features. Increased immune infiltration, inhibition of tumor related pathways, widespread genomic mutations and prone immunotherapy were observed in C3. Furthermore, a five immune genes signature was constructed and predicted overall survival for CESC, which successfully validated in GSE44001 dataset. A positive phenomenon was observed between five hub genes expressions and methylation. Similarly, high group enriched in matrix related features, while immune related gene signatures were enriched in low group. Immune cell, immune checkpoints genes expression levels were negatively, while most TME gene signatures were positively correlated with Risk Score. In addition, high group was more sensitive to drug resistance.

**Conclusion:** This work identified three distinct immune subtypes and a five genes signature for predicting prognosis in CESC patients, which provided a promising treatment strategy for CESC.

## 1 Introduction

Globally, there are more than 500,000 new cases of cervical cancer every year, and about 300,000 deaths from cervical cancer, and its incidence and mortality rank the fourth place in female malignant tumors (Bray et al., 2018). Large-scale promotion of HPV vaccination and early screening and diagnosis of cervical cancer has reduced the disease burden of patients to some extent. The traditional treatment, mainly surgery and supplemented by chemoradiotherapy, has a good effect on the treatment of early cervical cancer, but the 5-year survival rate of advanced, metastatic, and recurrent cervical cancer is less than 20% (Pfaendler and Tewari, 2016; Tewari et al., 2017).

Tumor microenvironment (TME) is the cellular environment in which tumor cells reside, which is composed of immune cells, mesenchymal cells, endothelial cells, inflammatory mediators, and extracellular matrix (ECM) (Hanahan and Weinberg, 2011; Hanahan and Coussens, 2012). The cells and molecules in TME are in a dynamic process that reflects the evolutionary nature of cancer and work together to promote immune escape, growth, and metastasis of tumors (Jiang et al., 2019; Ren et al., 2020). Immune cells and stromal cells are two major types of non-tumor components, which are considered to have important value in the diagnosis and prognosis of tumors (Zhu et al., 2021). In recent years, immunotherapy is a new means of tumor treatment. Its mechanism is to significantly improve the survival time by reactivating the anti-tumor immune system to strongly and continuously kill tumor cells. Currently, the most comprehensive immunotherapy is immune checkpoint inhibitor, whose representative drug is programmed death protein 1(PD-1) inhibitor (Pembrolizumab), which has been proved effective in a variety of cancers, but the overall objective effective rate is only 20%–30% (Iwai et al., 2017). Currently, the molecular targets used to guide immunotherapy are mainly limited to the expression level of programmed death protein ligand 1(PD-L1), high microsatellite instability (MSI-H) (Le et al., 2015), mismatch fixes system defects (dMMR) (Le et al., 2017), Tumor mutation burden (TMB) (Goodman et al., 2017; Yarchoan et al., 2017). TMB to predict the inaccurate treatment response of immunosuppressive agents in some cancer patients. Therefore, it is particularly important to screen more reasonable molecular markers to guide immunotherapy through comprehensive analysis of tumor microenvironment.

In view of this, this study obtained CESC expression profile data through The Cancer Genome Atlas (TCGA) database, and analyzed the relationship between immune pathway score and survival prognosis of patients with CESC by ssGSEA algorithm. Combined with the data set from the Gene Expression Omnibus (GEO) database (GSE44001), differentially expressed genes, (DEGs) analysis, functional enrichment and survival analysis were performed to screen out hub genes to construct prognostic models, and to explore the relevance of prognostic models in predicting the prognosis of patients with CESC and immunotherapy, so as to provide references for the research of biomarkers related to CESC immunity and immunotherapy.

## 2 Materials and methods

### 2.1 Data acquisition and preprocessing

Using “CESC”, “transcriptome profiling (transcripts per million (TPM))”, and “Gene Expression Quantification” as search terms, the results can be obtained from the TCGA database to download a sequence dataset containing 291 CESC tissues and corresponding clinical information. Using “cervical cancer” as a keyword in the GEO database. The gene-chip dataset GSE44001 contains 300 CESC tissues was downloaded.

For TCGA-CESC, the sample with clinical information, survival time greater than 0 and Status (alive and death) is retained and Ensembel is converted into Gene symbol, and the expression with multiple Gene Symbol is the median value. For the GSE44001 dataset, probes are mapped to genes based on annotation information, and probes that match one probe to multiple genes are removed. When multiple probes matched a gene, the mean value was taken as the expression value of the gene.

### 2.2 ssGSEA analysis

Twenty nine TME related gene signatures, covering known cellular and functional TME properties, were extracted from previously study (Bagaev et al., 2021). A total 257 genes were found in 29 gene signatures and ssGSEA using GSVA package (Yi et al., 2020) was employed to quantitate TME score.

### 2.3 Sample cluster analysis

ConsensusClusterPlus (Wilkerson and Hayes, 2010) was employed to construct consistency matrix for TCGA-CESC samples clustering on account on 29 TME gene signatures scores. 80% samples were carried out 500 bootstraps using km algorithm and distance of 1-pearson correlation. Number of Clusters was set as 2–10 and optimal cluster number was determined in terms of consistency matrix and cumulative distribution function. Principal component analysis (PCA) was also performed to test rationality of molecular subtype distribution.

### 2.4 Evaluation of immune infiltration

CIBERSORT algorithm (https://cibersort. stanford.edu/) was used to quantify the relative abundance of 22 types of immune cells in CESC. At the same time, the ESTIMATE (Yoshihara et al., 2013) software was used to calculate the proportion of immune cells.

### 2.5 Gene set enrichment analysis (GSEA)

All candidate gene sets in the KEGG database were used for GSEA (Subramanian et al., 2005) pathway analysis to identify unique biological process pathways in molecular subtypes, with FDR <0.05 considered to be significantly enriched. At the same time, the R software package GSVA was used for single sample GSEA analysis (ssGSEA), and the score of each sample on 26 biological pathways was calculated to obtain the ssGSEA score of each sample corresponding to each function. kruskal.test examines the differences between molecular subtypes.

### 2.6 Immunotherapy and chemotherapy

T-cell-inflamed gene expression profile (GEP) score of 18 genes (Ayers et al., 2017), Th1/IFNγ gene signature score (Danilova et al., 2019), combined genes from the published Th1 signature and genes from IFNγ signaling pathway from Reactome database, and cytolytic activity score (Gao et al., 2020) were calculated by ssGSEA to predicted clinical response to immune checkpoint blockade.

The expression levels of immune checkpoint genes, including immune activation genes and immune inhibition genes, were determined in molecular subtypes with kruskal. test (FDR< 0.05).

TIDE (Xiao et al., 2018; Fu et al., 2020) software was used to evaluate the potential clinical effects of immunotherapy included dysfunction of tumor infiltration cytotoxic T lymphocytes (CTL) (Dysfunction) and exclusion of CTL (Exclusion), M2 subtype of tumor-associated fibroblasts (CAF), tumor-associated macrophages (TAM), myeloid-derived suppressor cells (MDSCs), a higher TIDE predictive score indicates a greater likelihood of immune escape, suggesting that patients are less likely to benefit from immunotherapy.

pRRophetic (Geeleher et al., 2014) was used to predict the sensitivity of traditional medicines to half maximal inhibitory concentration (IC50).

### 2.7 Construction and validation of prognosis model

Among molecular subtypes, limma analysis (Ritchie et al., 2015) and univariate cox regression analysis were implemented to screen genes affecting CESC prognosis (*p* < 0.05). Random Forest SRC package was introduced to construct a random forest model and the most highly predictive variables were screened when variable importance (VIMP) value> 0.4. Finally, the optimal genes were used to constructed Risk score using stepAIC method in MASS package.

$$Risk\;score=\sum {coef}_{i}*Expi$$

Expi is the expression level of genes, and coefi is the regression correlation coefficient.

Survminer package was conducted to determine optimal cutoff to divided CESC samples into high group and low group. KM survival and ROC analysis using timeROC package were used to predict performance of Risk score. TCGA-CSEC was a training dataset and GSE44001 dataset was acted as independently validate dataset.

### 2.8 Statistical analysis

R (4.0.2) software was used for statistical analysis. WebGestaltR package (Yu et al., 2012) was used to carry out functional enrichment analysis. Genetic mutations were determined by maftools. Wilcoxon non-parametric rank sum test was used to analyze the differences. *p* < 0.05 was considered to be statistically significant. Sangerbox was used for analysis (Shen et al., 2022).

## 3 Results

### 3.1 29 TME gene signatures was association with clinical characteristics for TCGA-CESC samples

**FIGURE 1:**
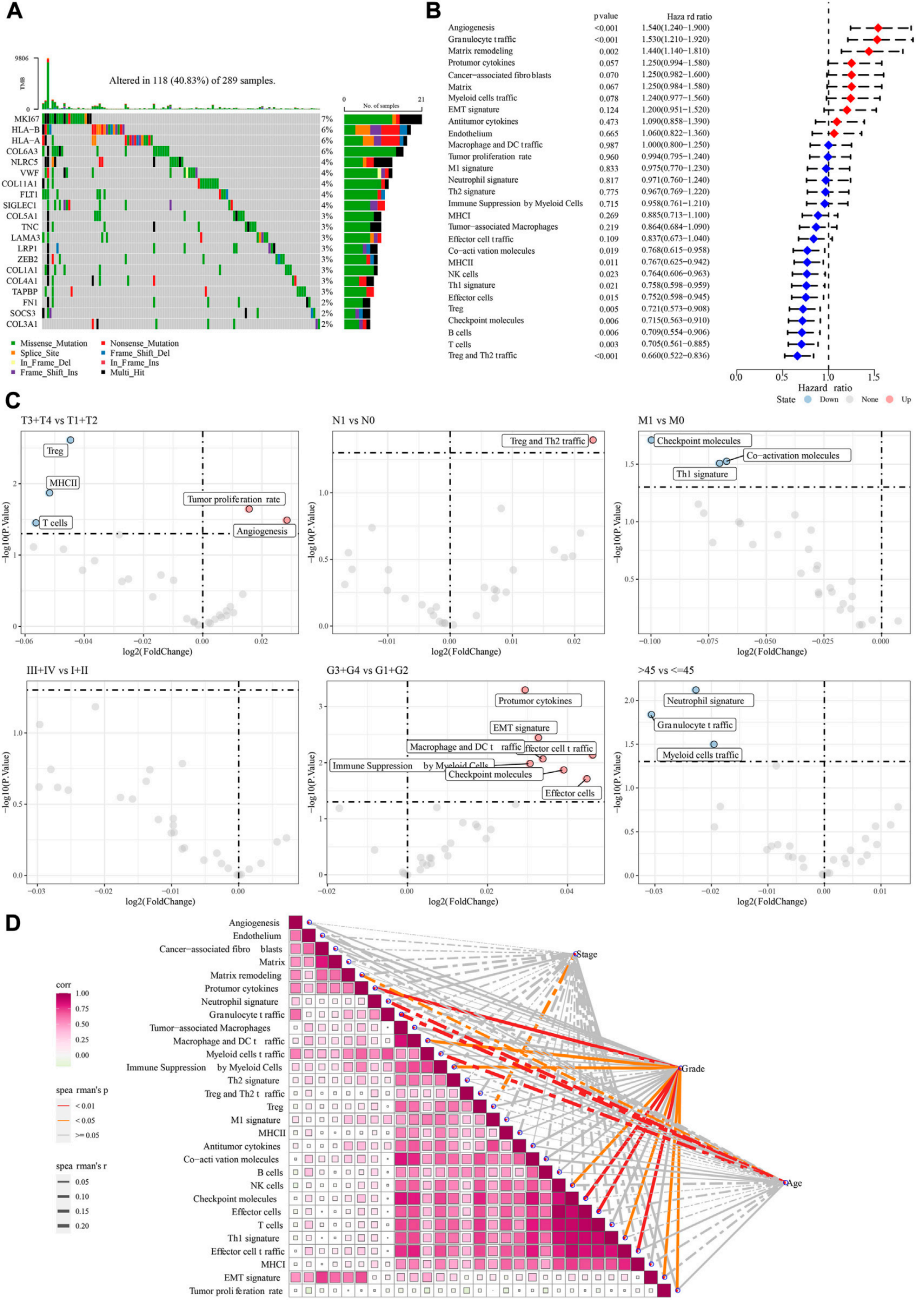
The association between 29 TME gene signatures and clinical characteristics in TCGA-CESC patients. **(A)**: Top20 TME related genes mutations in TCGA-CESC dataset. **(B)**: Univariate cox regression analysis of 29 TME gene signatures. **(C)**: The differences of 29 TME gene signatures among clinical feature grouping. **(D)**: Correlation between 29 TME gene signatures with each other as well as stage, grade, and age. *p < 0.05; **p < 0.01; ***p < 0.001; ****p < 0.0001; ns: no significance.

As we know, somatic mutations could lead to carcinogenesis. 199 of 257 genes (from 29 TME gene signature) were mutated and Top20 genes mutation rate were showed, among, MKI67 had highest mutation rate (7%) (Figure 1A). Univariate cox regression analysis of 29 TME gene signatures found 13 TME gene signatures affecting prognosis of CESC samples (Figure 1B). The differences of TME gene signatures scores in clinical features indicated that Tumor proliferation rate, Angiogenesis scores were increased in T3 + T4 stage, Protumor cytokines, Macrophage and DC traffic, Effector cell traffic, Immune Suppression by Myeloid Cells, Effector cells scores were enhanced in G3 + G4 stage (Figure 1C). TME gene characteristics were positively correlated with each other and with Grade (Figure 1D).

### 3.2 Identification of three molecular subtypes

**FIGURE 2:**
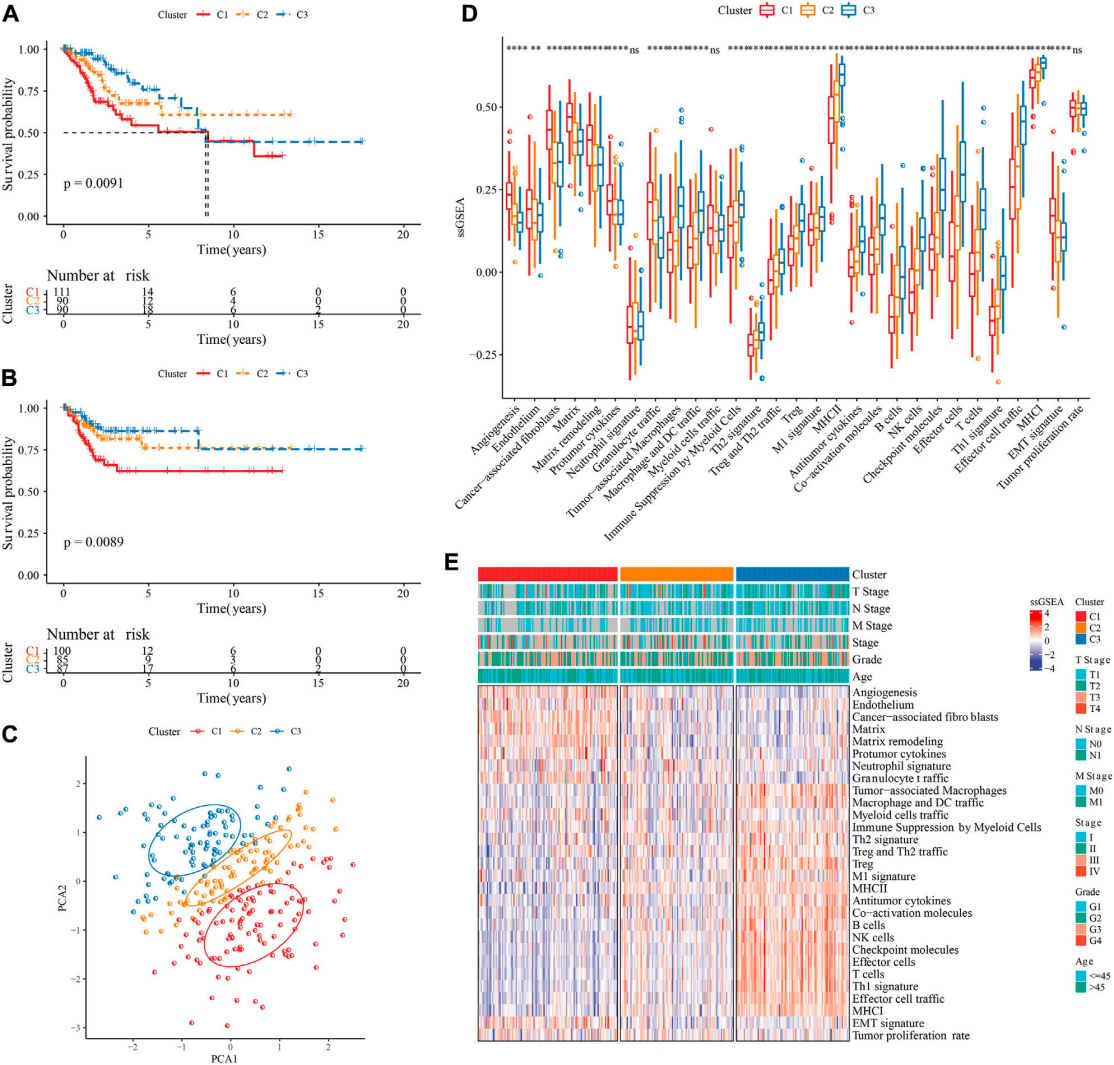
Identification of three molecular subtypes. **(A)**: KM curve of overall survival (OS) prognosis among three TME subtypes in the TCGA-CESC cohort. **(B)**: KM curve of progression-free survival (PFS) prognosis among three TME subtypes in the TCGA-CESC cohort. **(C)**: Principal component analysis of three TME subtypes. **(D)**: Statistical chart of the differences of 29 TME gene signatures among three TME subtypes. **(E)**: heatmap of differences of 29 TME gene signatures as well as clinical characteristics among three TME subtypes. *p < 0.05; **p < 0.01; ***p < 0.001; ****p < 0.0001; ns: no significance.

Based on 29 TME gene signature, TCGA-CESC samples were divided into three molecular subtypes when k = 3 on account of CDF and CDF delta area (Supplementary Figure S1A–C). KM survival curve showed that the overall survival and progression-free survival (PFS) in C3 had longest time, followed by C2 and C1 (Figures 2A, B). PCA suggested that the three molecular subtypes have distinct regional divisions (Figure 2C). The distribution of TME gene signatures among three molecular subtypes indicated that immune related gene signatures, such as Treg and Th2 traffic, Antitumor cytokines, were enriched in C3, while Matrix related gene signatures, such as Angiogenesis, Endothelium, Cancer-associated fibroblasts, Matrix, Matrix remodeling, were enriched in C1 (Figure 2D). TNM stage also had distribution differences among three molecular subtypes (Figure 2E).

### 3.3 Differences of infiltration of immune cells and somatic cell mutation among three molecular subtypes

**FIGURE 3:**
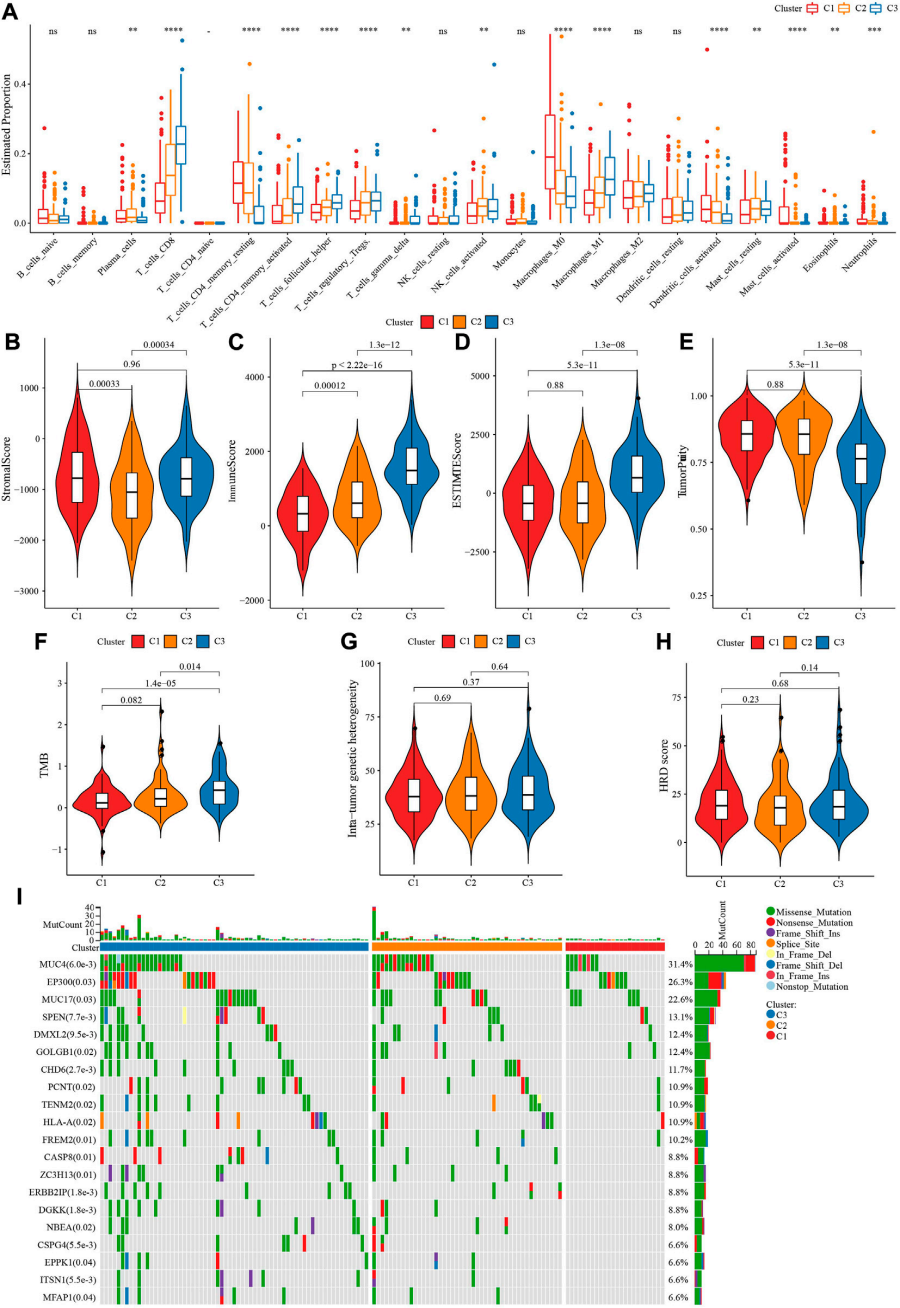
Immune infiltration analysis among three TME subtypes. **(A)** CIBERSORT analysis of 22 immune cells distribution among three TME subtypes. **(B)** The difference of StromalScore among three TME subtypes. **(C)** The difference of ImmuneScore among three TME subtypes. **(D)** The difference of ESTIMATEScore among three TME subtypes. **(E)** The difference of TumorPurity among three TME subtypes. **(F)** The difference of TMB among three TME subtypes. **(G)** The difference of Intra-tumor genetic heterogeneity among three TME subtypes. **(H)** The difference of HRD score among three TME subtypes. **(I)** Somatic cell mutation among three TME subtypes. ***p < 0.001; ****p < 0.0001.

In TCGA-CESC dataset, CIBERSORT analysis on 22 immune cells showed 17 of which had statistical significance among three molecular subtypes, such as T_cells_CD8, T_cells_CD4_memory_activated were involved in C3, while C1 enriched in T_cells_CD4_memory_resting, Macrophages_M0, Dendritic_cells_activated (Figure 3A). ESTIMATE analysis demonstrated that C3 had enhanced StromalScore, ImmuneScore, ESTIMATEScore, while TumprPurity was lowest (Figures 3B–E). Changes in genome among three molecular subtypes were explored, and we found that C3 harbored a significantly higher TMB (Figure 3F). No statistical significance of Mutant-allele tumor heterogeneity and HRD score among subtypes were observed (Figures 3G, H). In addition, MUC4, EP300, MUC17 genes had a wide range of somatic mutations in CESC (Figure 3I).

### 3.4 Functional characterization of three molecular subtypes

**FIGURE 4:**
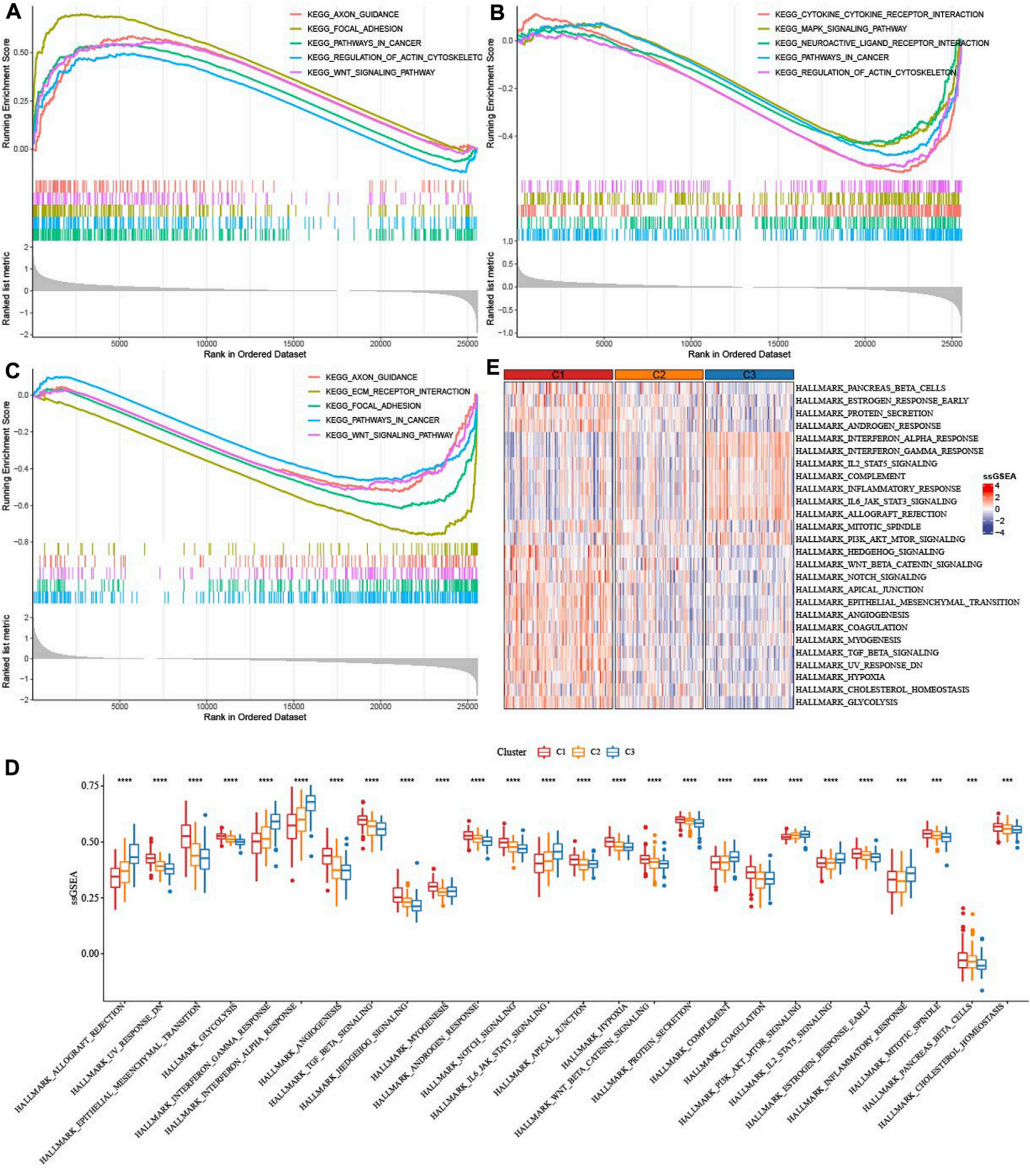
Enrichment of pathways. **(A)** GSEA analysis of five pathways were activated in C1. **(B)** GSEA analysis of five pathways were inhibited in C2. **(C)** GSEA analysis of five pathways were inhibited in C3. **(D,E)**: ssGSEA analysis of 26 pathways score distribution among three TME subtypes.

GSEA analysis indicated that axon guidance, focal adhesion, pathways in cancer, regulation of actin cytoskeleton, WNT signaling pathway were activated in C1 (Figure 4A), Cytokine-cytokine receptor interaction, MAPK signaling pathway, Neuroactive ligand receptor interaction, pathway in cancer were inhibited in C2 (Figure 4B), Axon guidance, ECM receptor interaction, pathway in cancer and WNT signaling pathway were inhibited in C3 (Figure 4C). ssGSEA analysis of 26 pathways scores had difference among three molecular subtypes. EMT-related pathways such as HALLMARK_WNT_BETA_CATENIN_SIGNALING were enriched in C1, in addition, the C3 subtype is significantly enriched in some immune-related pathways such as HALLMARK_INTERFERON_ALPHA_RESPONSE and HALLMARK_INTERFERON_GAMMA_RESPONSE (Figures 4D, E). Those data suggested that C3 presented immunoinfiltration state, and Cell growth-related pathways were activated in C1.

### 3.5 Analysis of immunotherapy and chemotherapy among three molecular subtypes

**FIGURE 5:**
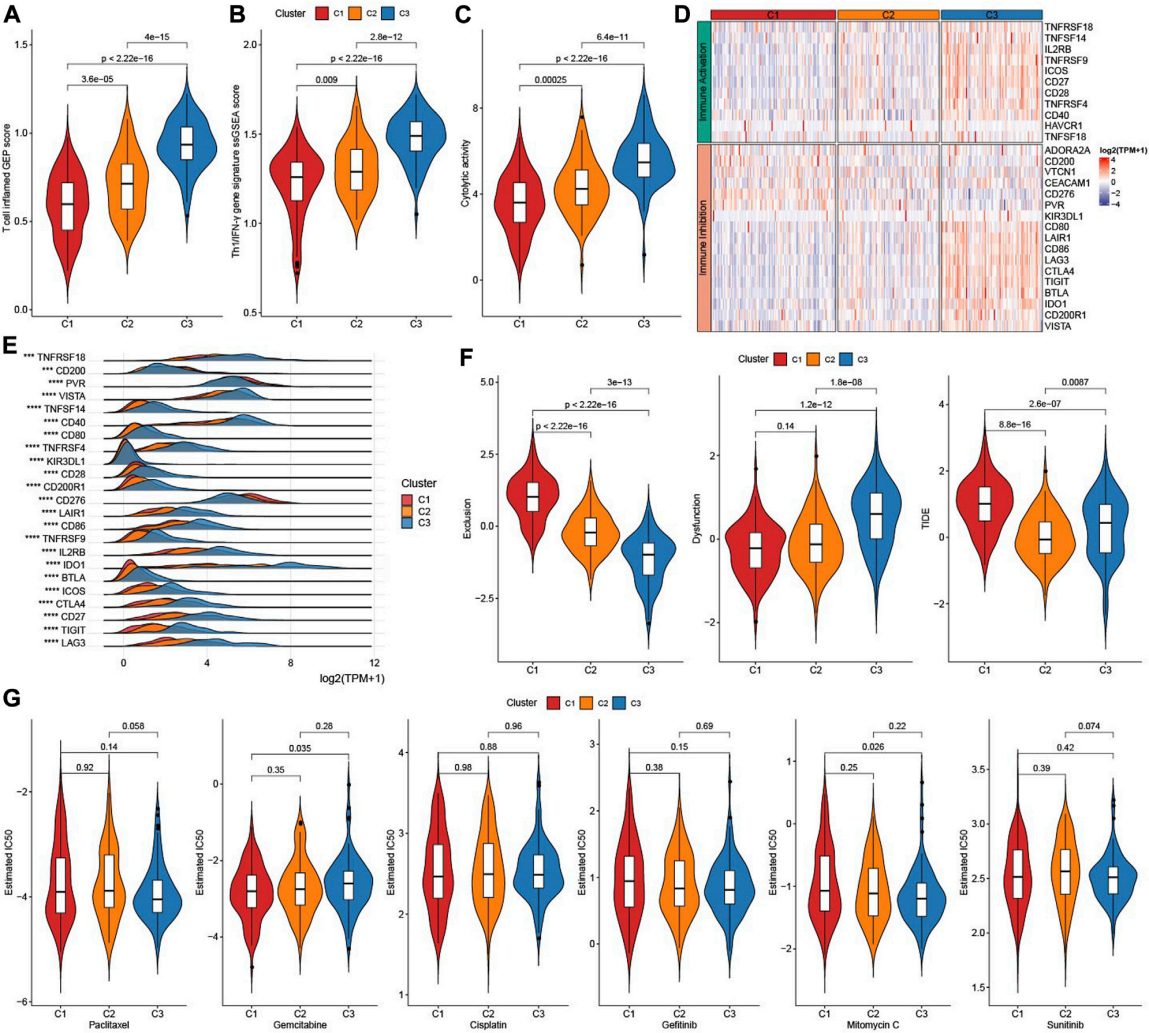
Immunotherapy analysis. **(A)** The difference of T cell inflamed GEP score among three TME subtypes. **(B)** The difference of Th1/IFNγ score among three TME subtypes. **(C)** The difference of Cytolytic activity score among three TME subtypes. **(D)** Heatmap of immune checkpoints genes among three TME subtypes. **(E)** the expressions of immune checkpoints genes among three TME subtypes. **(F)** TIDE analysis among three TME subtypes. **(G)** The box plots of the estimated IC50 for Paclitaxel, Gemcitabine, Cisplatin, Gefitinib, Mitomycin C, and Sunitinib among three TME subtypes.

As showed in Figures 5A, B, Figure 3 factors (T cell inflamed GEP score, Th1/IFNγ gene signature score, and Cytolytic activity score) that predict immunotherapy effect were all elevated in subtype C3 (Figures 5A–C). Given that immune checkpoint blockade (ICB) is a key factor for cancer immunotherapy, we evaluated a few representative genes. Most immune inhibition genes and activation genes were upregulated in C3 (Figure 5D). Moreover, 23 immune checkpoint genes had highest expressions in C3 (Figure 5E). Exclusion score and TIDE score were significantly highest in C1, while Dysfunction score was highest in C3 (Figure 5F). Sensitivity analysis of molecular subtypes to traditional chemotherapy drugs showed C3 was more sensitive to Paclitaxel, Mitomycin C, and C1 maybe benefit from Gemcitabine (Figure 5G).

### 3.6 Construction and validation of risk model

**FIGURE 6:**
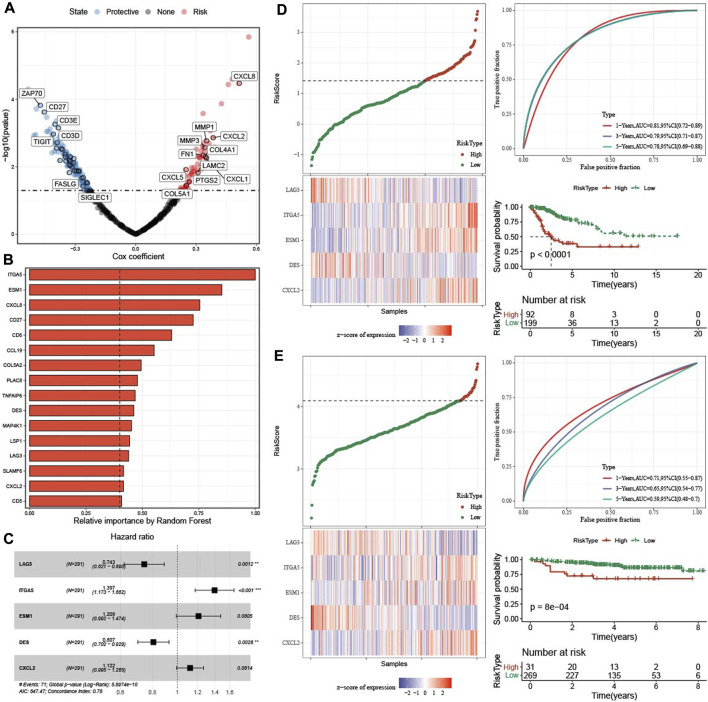
Construction and validation of prognosis model. **(A)** Univariate cox regression analysis of TME related genes. **(B)** The 16 most predictive genes selected by random survival forest. **(C)** Univariate cox regression analysis of five hub genes. **(D)** The distribution of RiskScore, expression of five hub genes in TCGA-CESC dataset. ROC analysis and AUC of RiskSore in TCGA-CESC dataset. KM survival curve of high group and low group in TCGA-CESC dataset. **(E)** The distribution of RiskScore, expression of five hub genes in GSE44001 dataset. ROC analysis and AUC of RiskSore in GSE44001 dataset. KM survival curve of high group and low group in GSE44001 dataset.

Firstly, DEGs were screened among three molecular subtypes, which 165 upregulated genes and 96 downregulated genes in C1 (Supplementary Figure S2A), 216 increased genes and 106 decreased genes in C3 (Supplementary Figure S2B). Finally, 429 DEGs were found among three molecular subtypes (Supplementary Figure S2C). 186 genes affecting prognosis of CESC samples were screened from 429 genes (Figure 6A). 186 genes were reduced to 16 genes using a random forest model (Figure 6B). Finally, five hub gene were determined from 16 genes by stepAIC method (Figure 6C). RiskScore = −0.297*LAG3 + 0.334*ITGA5+0.19*ESM1-0.214*DES + 0.115*CXCL2. The five hub genes expressions were positively correlated with methylation levels (Supplementary Figure S3).

In TCGA-CESC dataset, the distribution of RiskScore and five genes expression were showed. 1-, 3-, five- year AUC was 0.81, 0.79, and 0.78 respectively, and patients in high group had worse survival time (Figure 6D). In GSE44001 queue, the 1-, three-, and five- year AUC was 0.71, 0.65, and 0.59, respectively, and samples in high group also had poor survival time (Figure 6E).

### 3.7 Association clinical features and RiskScore

**FIGURE 7:**
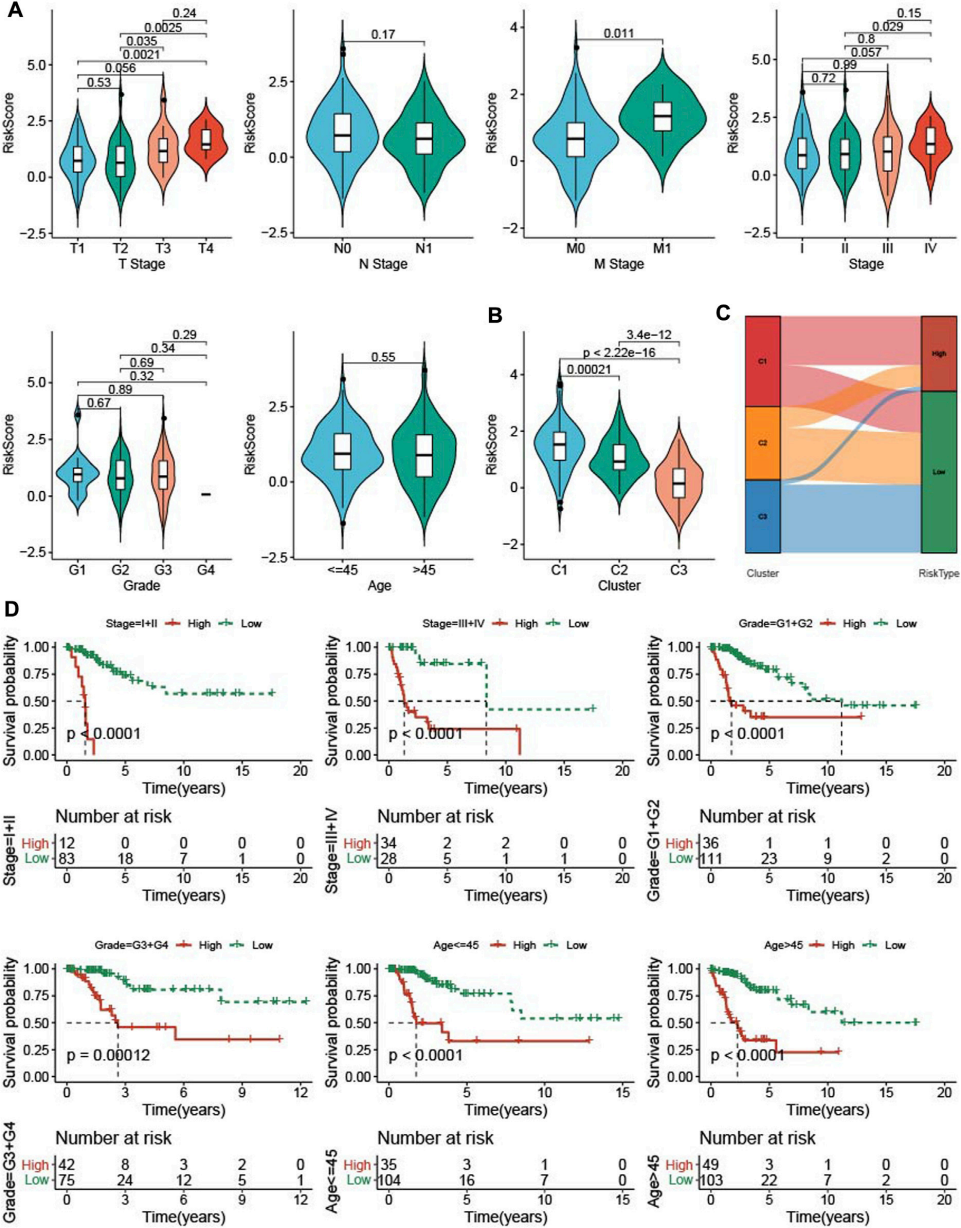
The RiskScore differences on samples with clinical features. **(A)** the differences of RiskScore in patients with various clinical features including T stage, N stage, M stage, stage, grade, and age. **(B)** the differences of RiskScore among three TME subtypes. **(C)** Matches of two subtypes and high- and low-groups. **(D)** KM survival of patients in high group and low group with various clinical features divided by RiskScore.

To know the relationship between RiskScore and clinical features, RiskScore was determined among clinicopathological features. The higher the clinical grade, the higher the RiskScore (Figure 7A). The C1 subtype with good prognosis has a higher RiskScore, while the C3 molecular subtype with a poor prognosis has the lowest RiskScore (Figure 7B), and most of the RiskScore-high samples were C1 patients (Figure 7C). samples with clinical features were divided into High group and low group based on RiskScore, KM curve demonstrated that patients in low group had a better prognosis (Figure 7D).

### 3.8 Characteristics of immunity of identified CESE subtypes

**FIGURE 8:**
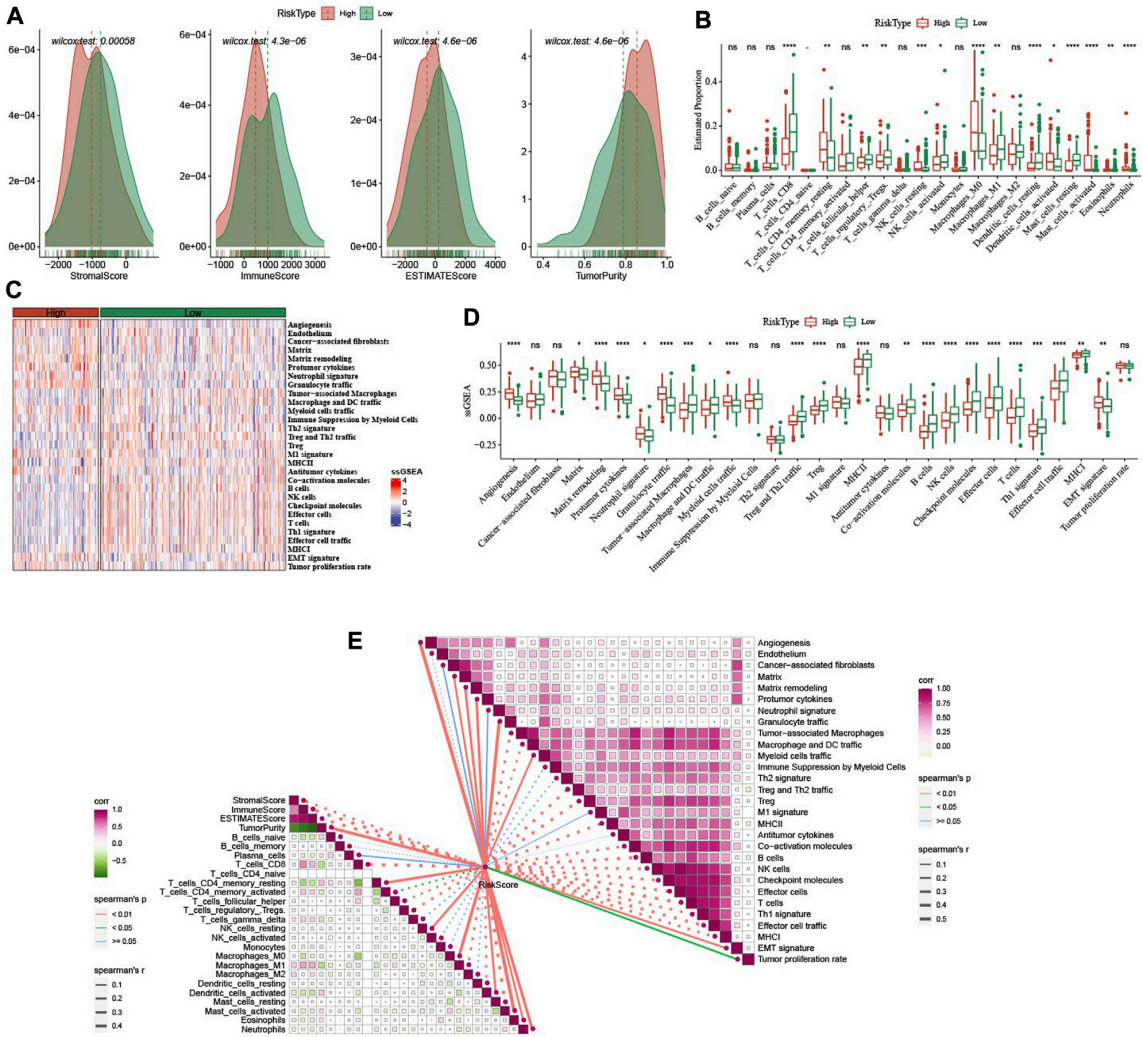
Immune infiltration analysis between high- and low-group. **(A)** ESTIMATE analysis between high- and low-group in TCGA-CESC dataset. **(B)** The distribution of 22 immune cells between high- and low-group in TCGA-CESC dataset. **(C,D)**: the differences of 29 TME gene signatures scores between high- and low-group. **(E)** The association analysis between RiskScore and immune features as well as 29 TME gene signatures. *p < 0.05; **p < 0.01; ***p < 0.001; ****p < 0.0001; ns: no significance.

In low group, StromalScore, ImmuneScore, and ESTIMATEScore were higher and TumorPurity was lower (Figure 8A). 14 of 22 immune cells score had significance between high group and low group (Figure 8B). In 29 TME gene signatures, 22 of which reach statistical difference between high group and low group (Figures 8C, D).

In addition, we also analyzed the relationship between RiskScore and immune infiltration and immune cells in 22. It was found that RiskScore was negatively correlated with StromalScore, ImmuneScore, ESTIMATEScore, T_cells_CD8, T_cells_follicular_helper, and Macrophages_M1. However, there were significant positive correlations with Angiogenesis, Matrix, Matrix remodeling, Protumor cytokines, Myeloid cells traffic (Figure 8E).

### 3.9 Immunotherapy response of identified CESE subtypes

**FIGURE 9:**
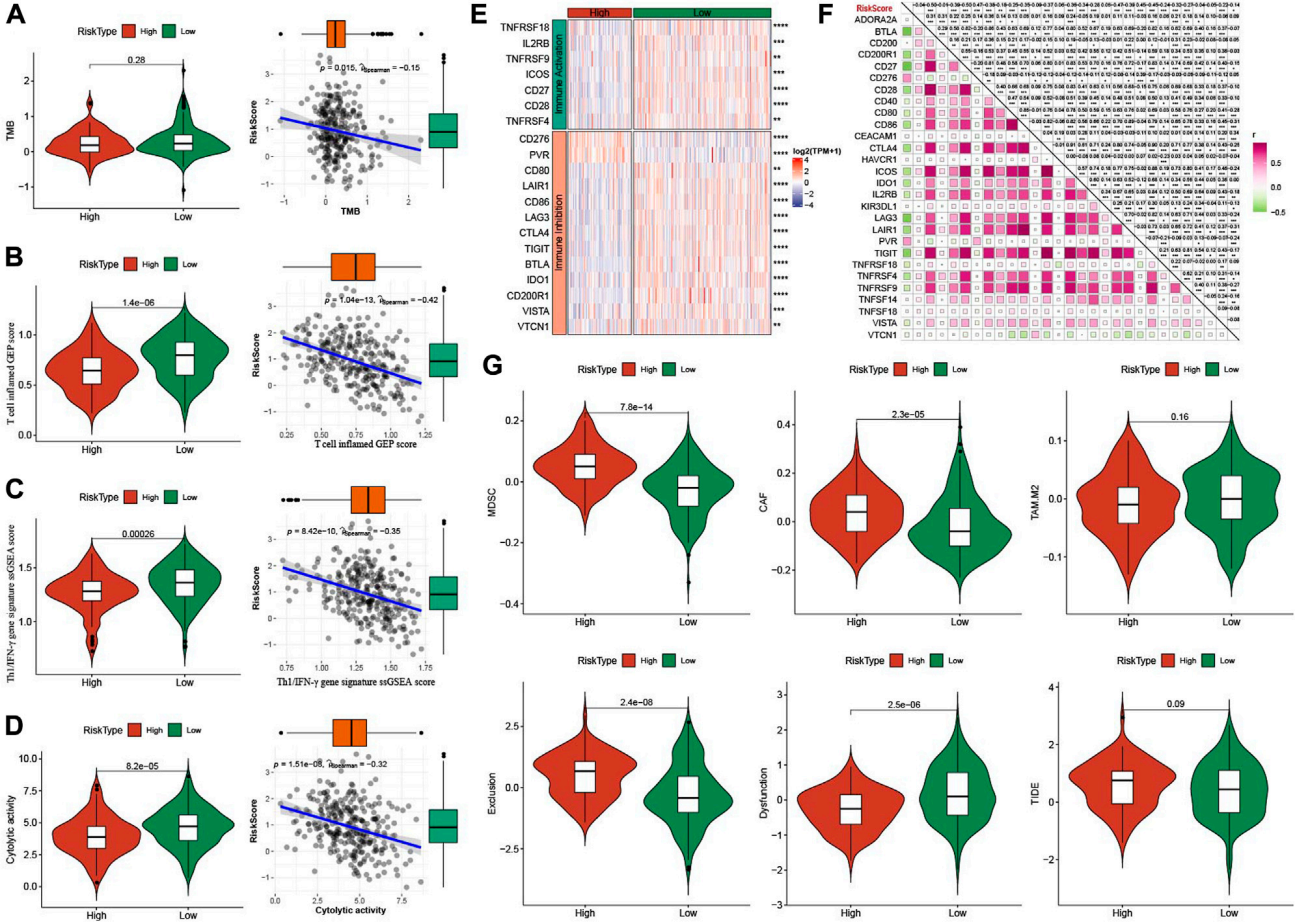
Analysis of immunotherapy between high- and low-group. **(A)** TMB differences between high- and low-group. The association between RiskScore and TMB. **(B)** T cell inflamed GEP score differences between high- and low-group. The association between RiskScore and T cell inflamed GEP score. **(C)** Th1/IFNγ gene signature score differences between high- and low-group. The association between RiskScore and T cell inflamed GEP score. **(D)** Cytolytic activity differences between high- and low-group. The association between RiskScore and Cytolytic activity. **(E)** The expression of immune checkpoints genes between high- and low-group. **(F)** The association between RiskScore and immune checkpoints genes. **(G)** TIDE analysis between high- and low-group. *p < 0.05; **p < 0.01; ***p < 0.001; ****p < 0.0001; ns: no significance.

Firstly, we compared the TMB in high group and low group, which it had no significance between the two groups (*p* = 0.28), but there was a negatively association between RiskScore and TMB (Figure 9A). T cell inflamed GEP score, TH1/IFNγ gene signature score and Cytolytic activity score were all enhanced in low group, and all them were negatively correlated with RiskScore (Figures 9B–D). Immune checkpoint genes were higher expressions in low group and negatively correlated with RiskScore (Figures 9E, F). MDSC, CAF, TAM.M2, and Exclusion were decreased, while Dysfunction was increased in low group in comparison to high group (Figure 9G).

## 4 Discussion

Studies have shown that CESC interstitium has a large number of immune cell infiltration (Dossus et al., 2013; Liu et al., 2020), Immune cell infiltration is believed to play an important role in the development of various malignant tumors (Hanahan and Coussens, 2012; Yoshihara et al., 2013), and immunotherapy has made great progress in the field of anti-tumor. In this study, it was found that in the 29 TME gene signatures, the higher the CESC pathological grade, the higher the infiltration of some TME gene signatures, and the infiltration abundance is related to the patient’s prognosis. Targeted therapy targeting these immune cells is expected to improve the patient’s prognosis.

In this study, based on 29 TME gene signatures, the TCGA-CESC cohort samples were divided into three immune subtypes (C1, C2, C3), which showed significant differences in prognosis, immune characteristics, pathway enrichment, gene mutation, and immunotherapy. C3 with good prognosis presented immunoinfiltration state, and cell growth-related pathways were activated in C1 accompanied by poor prognosis. Based on the three immune subtypes, the risk model was constructed by univariate Cox regression analysis and random survival forest model. We found that patients in the low-risk group had longer survival than those in the high-risk group, and there were significant differences in immunoinfiltration and immunotherapy.

In recent years, immune system therapies such as immune checkpoint inhibitors have shown remarkable effects in the field of anti-tumor. Studies have shown that highly mutated tumor genes can induce the production of a large number of neoantigens, which can activate immune cells and lead to a tumor-suppressing immune response (Büttner et al., 2019). MSI is closely related to the efficacy of tumor immunotherapy (Baretti and Le, 2018). Multiple studies have demonstrated that TMB, T cell inflamed GEP, TH1/IFN-γ, TIDE are emerging biomarkers for predicting the efficacy of tumor immunotherapy (Samstein et al., 2019). This study found that T cell inflamed GEP and TH1/IFγ scores were negatively correlated with RiskScore, and the low-risk group had a lower TIDE score. We speculated that patients in the low-risk group may benefit from immunotherapy.

Among the five key genes, ITGA5, ESMI, and CXCL2 were risk factors for the prognosis of CESC, while LAG3, and DES were protective factors. Multiple studies have shown that increased ITGA5 expression predicts poor prognosis of tumors, such as ovarian cancer (Gong et al., 2016), breast cancer (Xiao et al., 2018), and lung cancer (Zheng et al., 2016). CXCL2 expression level was closely related to lymph node metastasis and prognosis of cervical cancer patients (Zhang et al., 2018; Yang et al., 2021). Patients with high levels of LAG-3 peripheral t cells may suppress the antitumor response in a way that PD-1 or CTLA-4 blockers cannot overcome. LAG-3 has shown promise as a target in preclinical models, and drugs targeting LAG-3 are already in the early stages of clinical development, showing modest activity in unselected patient populations (Grosso et al., 2007; Brignone et al., 2009; Kraman et al., 2020). Two other genes including ESMI, and DES were little studied and their involvement in CESC remains largely unexplored, and more basic researches are needed to reveal their biological function in CESC.

There are some limitations in this study. First, it is necessary to verify the significance of hub genes in cancer tissues through experiments, such as RT-qPCR, IHC, and Western blot. Second, although our results show good predictive potential and clinical value of the five gene prognostic signature, prospective studies are needed to demonstrate the clinical application and prognostic value of this model in patients.

In this study, based on 29 TME gene signatures, we not only identified three subtypes and constructed a 5-key genes prognostic signature of CESC, which had a potential prognostic value. Those fundings maybe provided prognosis prediction and precision treatment for clinicians.

## Data Availability

The original contributions presented in the study are included in the article/Supplementary Material, further inquiries can be directed to the corresponding author.

## Supplementary material

SUPPLEMENTARY FIGURE S1 Cluster analysis. A: Cumulative distribution function. B: Delta area of Cumulative distribution function. C: sample clustering in TCGA-CESC dataset when k=3.

SUPPLEMENTARY FIGURE S2 Identification of Differentially expressed gene. A: Differentially expressed gene in C1 vs C2/C3. B: Differentially expressed gene in C3 vs C1/C2. C: Differentially expressed gene among 3 molecular subtypes.

SUPPLEMENTARY FIGURE S3 The association of 5 hub genes expressions and methylation level.
