# Supplementary figures and images for: Identification of immune related molecular subtypes and prognosis model for predicting prognosis, drug resistance in cervical squamous cell carcinoma
Source: Front Genet. 2023 Mar 17;14:1137995. doi: 10.3389/fgene.2023.1137995 (PMC10063826; doi:10.3389/fgene.2023.1137995)

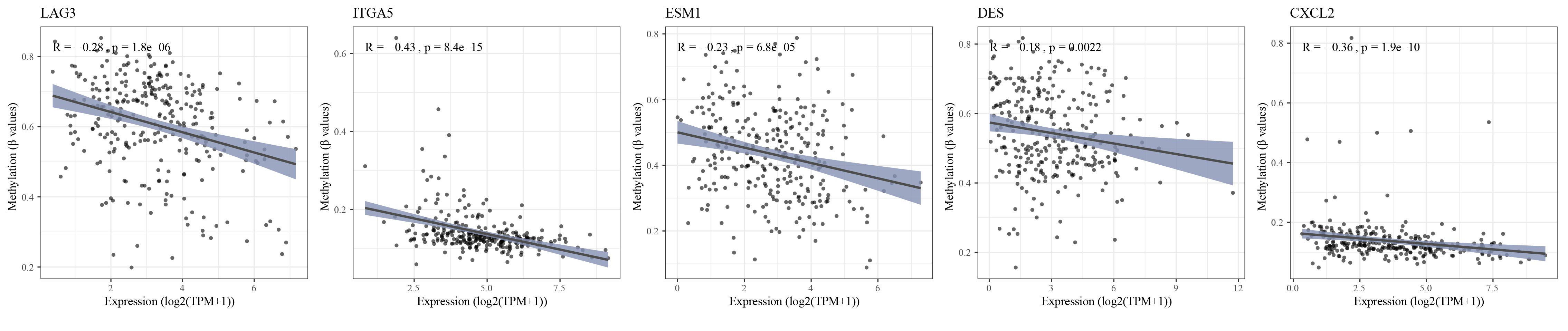

Supplement: Supplementary file 1 [file Image3.JPEG]

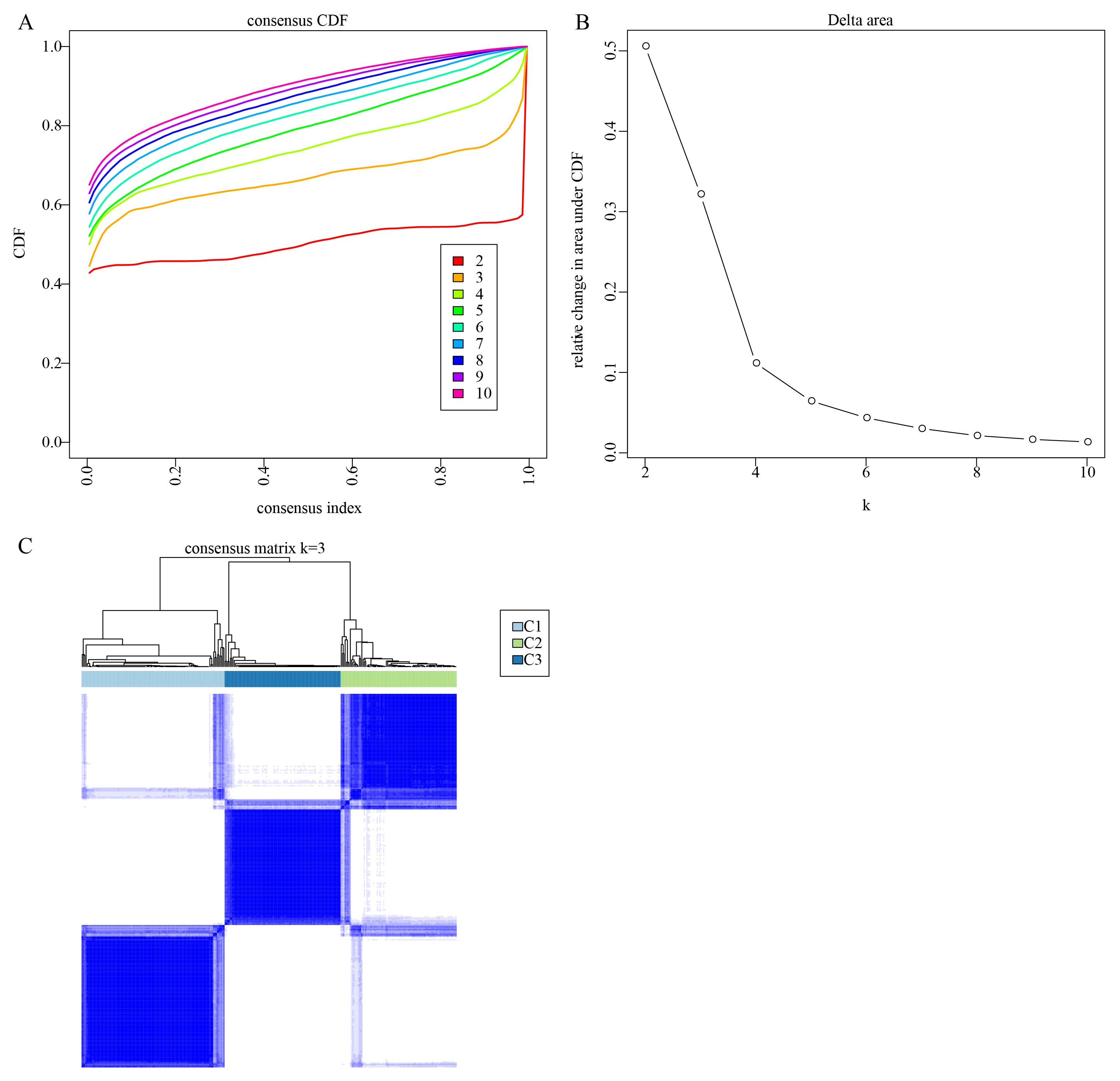

Supplement: Supplementary file 2 [file Image1.JPEG]

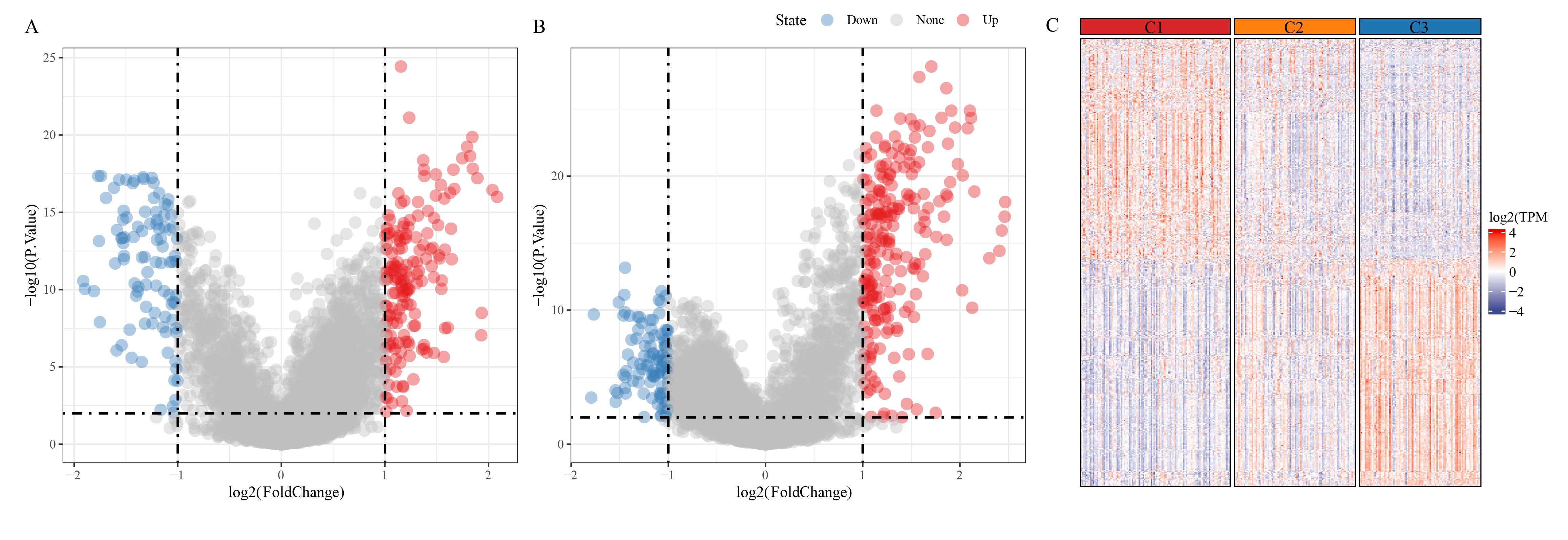

Supplement: Supplementary file 3 [file Image2.JPEG]
